# The 13-Valent Pneumococcal Conjugate Vaccine Elicits Serological Response and Lasting Protection in Selected Patients With Primary Humoral Immunodeficiency

**DOI:** 10.3389/fimmu.2021.697128

**Published:** 2021-07-05

**Authors:** Ailsa Robbins, Mathilde Bahuaud, Maxime Hentzien, Quentin Maestraggi, Coralie Barbe, Delphine Giusti, Richard Le Naour, Frederic Batteux, Amélie Servettaz

**Affiliations:** ^1^ Internal Medicine, Clinical Immunology and Infectious Diseases Department, University Hospital Centre, Reims, France; ^2^ Laboratory of Immunology, EA7509 IRMAIC, University of Reims Champagne-Ardenne (URCA), Reims, France; ^3^ Plateforme d’Immunomonitoring Vaccinal, Laboratory of Immunology, Cochin Hospital and University Paris-Descartes, APHP, Paris, France; ^4^ Clinical Research Department, EA3797, University of Reims-Champagne-Ardenne, Reims, France; ^5^ Laboratory of Immunology, Reims University Hospital, University of Reims Champagne-Ardenne, Reims, France

**Keywords:** primary humoral immunodeficiency, common variable immunodeficiency, IgG subclass deficiency, pneumococcal vaccine, conjugate vaccination

## Abstract

**Background:**

Patients with primary humoral immunodeficiency are more prone to invasive as well as recurrent pneumococcal infections. Therefore, anti-pneumococcal vaccination including the 13-valent conjugate vaccine is recommended. Nevertheless, to date, no data is available on immunogenicity of this vaccine in this population.

**Objective:**

To assess the immunogenicity and the persistence of protection up to one year after a 13-valent pneumococcal conjugate vaccine in patients with primary humoral immunodeficiency.

**Methods:**

Twenty-nine patients with common variable immunodeficiency or IgG subclass deficiency were vaccinated. Immune response and immune protection at baseline as well as at one, six and twelve months after vaccination were evaluated by measuring specific IgG serum concentrations (ELISA), and opsonophagocytic activities directed against selected pneumococcal (MOPA).

**Results:**

By ELISA, half of the patients had protective IgG concentrations before vaccination, 35.7% showed an immune response one month after vaccination, 71.4%, 66.7% and 56.0% of the patients were protected at one, six and twelve months respectively. Conversely, by MOPA, 3.4% of the patients were protected at baseline, 10.7% showed an immune response and 28.6%, 48.2% and 33.3% were protected at one, six and twelve months respectively. IgG subclass deficiency, Ig replacement therapy and higher IgG2 concentrations at diagnosis were associated with long-term protection.

**Conclusion:**

Pneumococcal conjugate vaccine improves immune protection and antibodies’ functionality in a subset of patients with primary immunodeficiency. Prime-boost vaccine strategy needs to be better and individually adapted.

## Introduction

Pneumococcal pneumonia is still a major cause of morbidity and mortality worldwide (1), particularly in children, older adults and subjects with comorbidities and was responsible for 300 000 deaths of children under the age of five and 690 000 deaths of adults over the age of 70, worldwide, in 2015. In France, anti-pneumococcal vaccination has been mandatory for infants before 2 months of age since 2018, and is recommended for immunocompromised or comorbid children and adults. Nevertheless, pneumococcal pneumonia still represented an estimated monthly incidence of 0.73 cases per 100.000 inhabitants in 2017 (and 2.08 cases per 100.000 in adults ≥65 years-old) (2). In adults, hospital stays due to pneumococcal pneumonia are long (average length of stay: 14.6 days) and in-hospital mortality is still high (8.8%) as shown by a recent study (3).

*Streptococcus pneumoniae* is a Gram-positive pathogen carried in upper airways in humans. Its capsule provides virulence, enabling the pathogen to invade organs. This capsule is composed of different polysaccharides, which are the basis for the classification of pneumococci into over 90 serotypes (4). Twenty-three of these serotypes are responsible for 80-90% of infections. Nowadays, two types of vaccines are available to prevent pneumonia and invasive pneumococcal diseases: a polysaccharidic vaccine and a conjugate vaccine. The polysaccharidic vaccine induces a T-independent response (implicating splenic marginal zone B- cells) against the twenty-three serotypes that are most implicated in human diseases (1, 2, 3, 4, 5, 6B, 7F, 8, 9N, 9V, 10A, 11A, 12F, 14, 15B, 17F, 18C, 19A, 19F, 20, 22F, 23F and 33F), while the conjugate vaccine elicits a T-dependent response (and therefore, gives rise to switched memory B-cell) against thirteen frequent serotypes (1, 3, 4, 5, 6A, 6B, 7F, 9V, 14, 18C, 19A, 19F, 23F).

Common variable immunodeficiency (CVID) and other antibody deficiencies like immunoglobin G subclass deficiency are the most frequent clinically significant primary immunodeficiencies (PID) in adults (5, 6). These immunodeficiencies are characterized by a significant decrease in serum immunoglobulin concentrations and poor vaccine response. Consequently, patients are more prone to bacterial infections and particularly pneumonia, that are eight times more frequent in CVID patients than in healthy population (7).

Based on data in healthy subjects, French and international guidelines recommend vaccinating humoral immunocompromised adults with the 13-valent conjugate vaccine (PCV13) followed by a polysaccharidic 23-valent vaccine (PPSV23) at least two months later (8, 9) although there is no data concerning immunogenicity of PCV13 in this population to date. Patients with humoral immunodeficiency usually develop poor and short-time vaccine response (10–12). This original study was therefore conducted to assess the immunogenicity and the persistence of protection up to one year after PCV13 in this population.

## Patients and Methods

### Study Population

Twenty-nine patients were enrolled from 2013 to 2016 at the University Hospital Centre in Reims, France. Patients were included if (1) they had CVID or IgG subclass deficiency as defined by the European Society for Immunodeficiency (ESID, see below), (2) had never received any anti-pneumococcal conjugate vaccine, (3) had not received any anti-pneumococcal polysaccharidic vaccine in the last three years and (4) if treatment was stable 6 months before enrolment and 12 months after inclusion for patients undergoing immunoglobulin replacement therapy as pneumococcal antibodies concentrations might vary from manufacturer to manufacturer but are stable from lots to lots (13–15) in Ig products.

CVID and IgG subclass deficiencies were defined according ESID criteria (16):

- CVID: marked decrease of IgG (≤ 5 g/L) and marked decrease in at least one of the isotypes IgM (≤ 0.3 g/L) or IgA (≤0.7 g/L)
- IgG subclass deficiency: reduction of at least 2 standard deviations in one or more IgG subclass (IgG1 ≤ 4.2 g/L, IgG2 ≤ 1.02 g/L, IgG3 < detection level) with or without IgA deficiency (≤ 0.7 g/L)

### Vaccination

At inclusion, patients received one single 0.5 ml intramuscular dose of 13-valent anti-pneumococcal conjugate vaccine (PCV13, Prevenar13^®^; Pfizer) following a routine visit. The vaccine contained polysaccharides from the pneumococcal serotypes 1, 3, 4, 5, 6A, 6B, 7F, 9V, 14, 18C, 19A, 19F and 23F, individually conjugated to a nontoxic diphtheria toxin cross-reactive material CRM197 protein. Blood-samples were obtained at baseline and one, six and twelve months after vaccination. Each blood sample was drawn before each infusion of immunoglobulin replacement therapy for patients undergoing such treatment.

### Flow Cytometric Analysis of Peripheral Blood Lymphocytes

Peripheral blood mononuclear cells (PBMCs) were stained for 15 minutes at room temperature with a mixture of the following antibodies: anti–CD27-APC, anti-CD45-FITC, anti-IgD-PE, anti-IgM-PERCP-Cy55, anti-CD19-V450 DAPI, anti–CD3 V500-Am-Cyan, anti-CD4-V450 DAPI, anti-CD8-APC H7, antiCD45RA-PE Cy7. Data acquisition was performed with FACSCanto II, FACS Lyric (BD Biosciences). Data were analyzed with the FACSDiva analysis software or FACSuite (BD Biosciences).

CVID patients were categorized according to the Euroclass classification based upon the percentage of circulating CD19^+^ B cells (B+: >1%, B-: ≤1% of total lymphocyte count). Among B+ category, patients were identified as smB+ if CD19^+^IgD^-^CD27^+^ smB cells represented more than 2% of circulating B-cell, or smB- (≤2%) (17).

### Serological Evaluation

All serological evaluations were done at the French Referral Laboratory for Anti-Pneumococcal Serology and Opsonophagocytosis activity (Cochin Hospital Center, Paris) (18). Standardized protocols issued from the pneumococcal reference laboratory at University of Alabama at Birmingham were used [*www.vaccine.uab.edu*] (19).

### Enzyme-Linked Immunosorbent Assay (ELISA)

IgG antibody concentrations for eight serotypes targeted by both PCV13 and PPSV23 (4, 6B, 7F, 9V, 14, 18C, 19F, and 23F) and two targeted only by PPSV23 (10F and 15B) were determined using a modified ELISA, as previously described (19, 20): Plates (Corning, Inc., Corning, NY) were coated with a serotype-specific pneumococcal PS antigen (American Type Culture Collection, Manassas, VA). Reference sera (007sp), control sera, or patient specimens were pre-absorbed with 5 µg/mL pneumococcal C-polysaccharide (Statens Serum Institut, Copenhagen, Denmark) and 10 µg/mL serotype 22F capsular polysaccharide (American Type Culture Collection). Anti-pneumococcal antibody levels were determined in each specimen by analysis of linear regression plots compared with the reference serum (007sp) (National Institute for Biological Standards and Control) (NIBSC). For each serotype, the geometric mean concentrations (GMC) and the corresponding 95% confidence intervals (CI) were calculated.

### Multiplex Opsonophagocytic Assay (MOPA)

Opsonization of the bacterial capsule by complement protein, facilitates its destruction by the phagocytic cell engaged in the immune response. This immunological response can be measured *ex-vivo*, by quantifying maximum dilution of patients’ serum capable of killing 50% of pneumococcal strain, in presence of complement and differentiated HL60 cells. Thus, opsonophagocytic assay allows us to evaluate functional immunogenicity of IgG and IgM specific serotype antibodies. Opsonophagocytic activities of antibodies for seven serotypes (4, 6B, 9V, 14, 18C, 19F and 23F) were measured by MOPA as previously described (19, 21). All serum samples were incubated at 56°C for 30 min before being tested. Sera were serially diluted in round-bottom 96-well plates (Corning Inc., Corning, NY). Frozen aliquots of target pneumococci were thawed, washed twice, diluted to a bacterial density of ∼50,000 CFU/ml, and added to the plates. After 30 min of incubation at room temperature with shaking at 700 rpm, complement and HL60 cells (ATCC) that had been differentiated to phagocytes were added to each well. Plates were incubated in a tissue culture incubator (37°C, 5% CO2) with shaking at 700 rpm. After a 45-min incubation, plates were placed on ice for 20 min. Ten μl of each well were spotted on to four different Todd-Hewitt broth-yeast extract agar plates. After application of an overlay agar containing one of four antibiotics to each agar plate and overnight incubation at 37°C, the number of bacterial colonies in the agar plates was enumerated.

Opsonization titers (OT) were defined as interpolated reciprocal serum dilution that kills 50% of the bacteria in the assay. The assay sensitivity is the lowest dilution of sera tested (limit of detection: LOD), which is normally 4 for undiluted sera, and is the same for each serotype. However, to quantify functional antibodies with more precision, the lower limit of quantification (LLOQ) was determined for each serotype-specific assay during assay validation. The LLOQs for each serotype were: *4*: 24, *6B*: 132, *9V*: 39, *14*: 85, *18C*: 47, *19F*: 74, *23F*: 30. Titers higher than the LLOQs were considered accurate and their values were reported. Titers below the LLOQs were set to a value of 2 (half a LOD) (22).

For each serotype, the geometric mean titers (GMT) and the corresponding 95% CI were calculated.

### Definition of Response and Protection Related to PCV13 Vaccination

Based on studies defining criteria for pneumococcal vaccine immune response and on previous works in immunocompromised patients, we used the following definition for response and protection (15, 18, 19, 23):

- Response towards a serotype was defined as a two-fold increase in serotype specific IgG concentration by ELISA or as a four-fold increase in OT from baseline by MOPA for the serotype. Patients developing a response for at least five of the tested serotypes were considered as “**global responders”** to the vaccine.
- Protection toward a serotype was defined by an IgG-concentration ≥1 µg/mL by ELISA or as at least an opsonization titer ≥LLOQ by MOPA for the serotype. Patients developing a protection for at least five of the tested serotypes were considered as “**globally protected”**.

### Statistical Analyses

Qualitative data were expressed in numbers and percentage, compared with chi square test when possible, or with Fisher’s exact test, as appropriate. Quantitative data were expressed in mean with their standard deviation and compared with Mann-Whitney U test. When needed, c-statistic was computed. All statistical analyses were performed using SAS version 9.4 (SAS Institute Inc., Cary, North Carolina, USA. A p-value below 0.05 was considered statistically significant. Figures were performed using GraphPad Prism version 7.03 (GraphPad Software).

### Ethical Approval

Study protocol was approved by our institutional review board in October 2014 and we received written and informed consent from every patient.

## Results

### Patients’ Characteristics

The demographical and immunological characteristics of the population are presented in **Table 1**. Fifteen patients were diagnosed with CVID (51.7%; patients n°1 to 15) and 14 patients with IgG subclass deficiency (48.3%; patients n° 16 to 29). The two groups were comparable except for sex. Mean IgG, IgA and IgM seric concentrations at diagnosis were significantly lower in the CVID group (IgG: 3.0 ± 1.7 *vs* 5.4 ± 0.6 g/L, p<0.0001; IgA: 0.4 ± 0.5 *vs* 1.0 ± 0.5 g/L, p<0.0004; IgM: 0.6 ± 0.8 *vs* 0.8 ± 0.5 g/L, p=0.02) as well as the number of switched memory B-cells (6.7 ± 8.2 *vs* 23.6 ± 18.3.10^6^/L, p=0.007).

**Table 1:** PID patients’ demographical and immunological characteristics.

ID types	N°	Sex	Age	Age at diagnostic	Genetic	Ig seric concentration (g/L)							Lymphocytes subpopulation (.10^6^/L)									Euroclass	
						IgG	IgG1	IgG2	IgG3	IgG4	IgA	IgM	Lymphocytes	B cell	% B cell	Naïve B cell	non smB	smB	% smB	T CD4 cell	Naïve T CD4^+^ cell	B	smB
**Common Variable ImmunoDeficiency**	**1**	M	21.2	19.2	NR	4.47	3.67	1.04	0.33	0.01	0.34	0.35	1120.00	146.00	**13.0**	123.60	14.60	3.40	**2.3**	348.20	89.90	**B+**	**smB+**
	**2**	M	53.6	43.9	April htz	1.15	1.00	0.00	0.25	0.00	0.11	0.00	1550.00	94.00	**6.1**	74.00	10.00	4.00	**4.3**	560.00	133.00	**B+**	**smB+**
	**3**	F	39.3	32.5	TACI htz	3.64	2.81	0.57	0.21	0.00	0.13	3.24	2100.00	59.00	**2.8**	7.00	40.00	12.00	**20.3**	1048.00	527.00	**B+**	**smB+**
	**4**	F	44.5	40.1	NR	5.3	4.12	1.08	0.14	0.06	0.26	1.29	2010.00	60.30	**3.0**	20.10	34.20	6.00	**10.0**	1065.10	542.60	**B+**	**smB+**
	**5**	F	19.4	4.2	NFKB2	1.58					0.06	0.21	3900.00										
	**6**	M	48.1	47.1	NR	4.76	3.57	1.31	0.19	0.12	0.38	0.30	910.00	136.40	**15.0**	118.20	9.10	2.70	2.0	354.50	63.60	**B+**	smB-
	**7**	F	16.0	15.9	NR	2.96	2.68	0.29	0.04	0.07	0.26	0.91	1920.00	421.50	**22.0**	344.80	57.50	0.00	0.0	823.80	229.90	**B+**	smB-
	**8**	M	66.0	52.5	APRIL	1.94	1.92	0.08	0.10	0.05	1.83	0.23	1240.00	148.00	**11.9**	74.00	40.00	31.00	**20.9**	423.00	102.00	**B+**	**smB+**
	**9**	M	42.5	14.1	NM	4.75					0.00	0.26	1210.00	92.00	**7.6**	82.00	7.00	1.00	1.1	520.00	222.00	**B+**	smB-
	**10**	F	24.2	24.1	NR	1.42	1.26	0.18	0.15	0.02	0.26	0.21	2140.00	213.60	**10.0**	192.20	10.70	0.00	0.0	832.90	128.10	**B+**	smB-
	**11**	F	55.1	55.1	NR	4.88	3.07	1.65	0.34	0.01	0.68	0.53	990.00	128.40	**13.0**	118.60	3.00	6.90	**5.4**	405.10	29.60	**B+**	**smB+**
	**12**	F	57.5	42.9	NM	0.68					0.06	0.35	1810.00	343.00	**19.0**	268.00	65.00	3.00	0.9	831.00	173.00	**B+**	smB-
	**13**	F	32.2	17.7	NM	0.00					0.00	0.11	1740.00	253.00	**14.5**	205.00	46.00	1.00	0.4	1098.00	89.00	**B+**	smB-
	**14**	F	47.9	47.0	NR	3.79	3.22	0.72	0.20	0.13	1.40	0.22	1340.00	201.40	**15.0**	181.30	5.40	12.10	**6.0**	698.20	402.80	**B+**	**smB+**
	**15**	M	53.3	45.6	NM	3.70					0.23	0.41	3040.00	548.00	**18.0**	471.00	55.00	11.00	**2.0**	1367.00	178.00	**B+**	**smB+**
**IgG Subclass Deficiency**	**16**	F	62.7	58.8	NR	5.28	3.88	0.86	0.18	0.16	0.71	1.76	2900.00										
	**17**	F	22.6	21.7	NR	5.57	4.88	0.09	0.17	0.11	0.91	0.73	1700.00	514.20	**30,2**	462.80	17.10	17.10	**3,3**	617.10	325.70		
	**18**	F	33.0	32.3	NR	4.99	2.94	1.37	0.35	0.21	1.45	0.94	1970.00	177.40	**9,0**	98.60	39.40	39.40	**22,2**	1005.20	374.50		
	**19**	F	35.4	20.8	NFKB2	5.30	3.23				0.39	0.32	3200.00										
	**20**	F	63.1	58.4	NR	6.42	3.76	2.19	0.20	0.28	1.51	0.72	1030.00	51.60	**5,0**	40.20	5.20	4.60	**8,9**	691.40			
	**21**	M	21.1	11.7	NR	5.87	2.89	2.22	0.26	0.07	0.38	0.66	2880.00										
	**22**	F	42.9	32.2	TACI htz	4.83	3.27	1.20	0.38	0.20	0.99	1.03	1550.00	87.00	**5,6**	58.00	7.00	14.00	**16,1**	851.00	419.00		
	**23**	M	53.9	45.4	NM	4.68	3.64	1.76	0.66	0.25	0.95	0.62	2170.00	192.00	**8,8**	102.00	29.00	46.00	**24,0**	1175.00	554.00		
	**24**	F	54.5	53.4	NR	4.80	3.24	1.63	0.34	0.07	1.85	1.20	2500.00	255.00	**10,2**	22.95	5.10	2.55	1,0	893.00			
	**25**	F	69.1	62.3	NM	4.96	3.68	0.69	0.07	0.10	0.41	0.22	900.00	225.80	**25,1**	167.10	33.40	19.90	**8,8**	433.60	122.90		
	**26**	F	54.6	52.9	NR	6.38	3.58	2.20	0.17	0.36	1.10	0.43	1880.00	262.90	**14,0**	165.30	37.60	52.60	**20,0**	995.30	169.00		
	**27**	F	65.4	57.6	NR	5.69	4.58	0.97	0.33	0.08	1.15	0.33	640.00	41.80	**6,5**	36.00	1.90	5.10	**12,2**	282.70	122.10		
	**28**	F	53.2	37.1	NR	5.35	3.11	1.94	0.26	0.04	0.58	0.89	1200.00										
	**29**	F	36.3	33.1	NR	5.85	3.83	1.66	0.19	0.33	1.26	1.95	1580.00	126.40	**8,0**	44.20	44.20	34.70	**27,5**	742.40	379.10		

Subclass, Isolated IgG subclass deficiency; CVID' Common Variable Immuno Deficiency.

Genetic: genetic variant tested among a predetermined genes panel (APRIL, TACI, BCMA, BAFF-R, BTK, CD40L, SAP), as part of a national study (DEFI) led by the national referral center for primary immunodeficiency (CEREDIH); NR, not realized; NM, No mutation.

Ig seric concentration; immunoglobulin seric concentration, at diagnosis when available, or before immunoglobulin substitution initiation. Lymphocytes subpopulation: immunophenotyping of the main B and T cell subpopulations in serum, when available (at any time during follow-up). Total count, total lymphocyte count; B cell, CD19+ cells; Naïve B cells, CD19+IgD+CD27-; non smB: non-switched memory B cells, CD19+IgD+CD27+; smB: switched memory B cells, CD19+IgD-CD27+; Naïve T CD4+ cells: CD4+CD45RA+.

Euroclass: B: CVID patients were classified as B+ if CD19+ B cells represented at least 1% of total lymphocyte count; smB: CVID patients were classified as smB+ or smB- depending on the percentage of smB cells among B cell (≤2%: smB-; >2%: smB+).

Bold characters were used to differentiate values in % from values in .10.6/L and to highlight patients in B+ and smB+ class.

As presented in **Table 2**, twenty-three of the subjects (79.3%) were regularly undergoing immunoglobulin replacement therapy. For three patients (10.3%) a past pneumococcal disease (two pneumonia and one bacteremia) had been documented. Seven CVID patients and two with IgG subclass deficiency had previously received a polysaccharidic anti-pneumococcal vaccination (PPSV23), at least three years before study entry.

**Table 2:** PID patients’ clinical characteristics.

ID types	N°	Ig replacement therapy	Prophylactic antibiotic	Prior PPV23 vaccination	Prior pneumo coccal infection	Infectious complications	Opportunistic infections	Bronchiectasis	Auto-immunity	Granuloma	Neoplasia
**Common Variable ImmunoDeficiency**	**1**	N	N	N	N	ENT	N	N	ITP	Y	N
	**2**	Y	Y	Y	N	ENT. LRTI	N	Y	ITP. AIHA	Y	N
	**3**	N	N	Y	N	N	N	N	N	N	N
	**4**	Y	Y	Y	N	ENT	N	N	N	N	N
	**5**	Y	N	Y	Pneumonia	ENT. LRTI	N	N	N	N	N
	**6**	Y	N	Y	N	ENT. LRTI	N	N	N	N	N
	**7**	Y	N	N	N	ENT	N	N	N	N	N
	**8**	Y	N	Y	N	ENT. LRTI	N	N	N	N	Skin
	**9**	Y	N	Y	N	ENT. LRTI	N	Y	N	Y	N
	**10**	Y	Y	N	N	ENT. LRTI	N	Y	N	N	N
	**11**	N	Y	N	N	N	N	Y	AIHA	Y	N
	**12**	Y	Y	N	N	ENT. LRTI. digestive parasitosis	N	N	N	Y	Skin
	**13**	Y	Y	N	N	LRTI	N	Y	ITP	N	N
	**14**	N	N	N	N	N	N	N	N	N	N
	**15**	Y	N	N	Bacteraemia	LRTI	N	N	ITP	N	Pancreas
**IgG Subclass Deficiency**	**16**	Y	N	N	N	ENT. onychomycosis	N	N	N	N	N
	**17**	N	N	N	N	N	N	N	Inflammatory rheumatism	N	N
	**18**	Y	N	N	N	ENT. LRTI	N	N	N	N	N
	**19**	Y	Y	Y	Pneumonia	ENT. LRTI	N	Y	Thyroiditis	N	N
	**20**	Y	N	N	N	LRTI	N	Y	N	N	N
	**21**	Y	Y	Y	N	ENT	N	N	N	N	N
	**22**	Y	Y	N	N	LRTI	N	N	N	N	Breast
	**23**	Y	N	N	N	ENT	Pulmonary tuberculosis	N	N	N	Skin
	**24**	N	N	N	N	N	N	N	N	N	N
	**25**	Y	N	N	N	N	N	N	N	N	N
	**26**	Y	Y	N	N	ENT	N	N	N	Y	N
	**27**	Y	N	N	N	ENT	N	N	N	N	N
	**28**	Y	N	N	N	ENT	N	N	N	Y	Breast
	**29**	Y	Y	N	N	ENT	N	N	N	N	N

F, female; M, Male; Y, Yes; N, No; ENT, ear, nose and throat; LRTI, lower respiratory tract infection; ITP, immune thrombocytopenic purpura; AIHA, auto-immune hemolytic anemia; Granuloma, granuloma proven by any tissue biopsy and/or splenomegaly and/or benign adenopathy.

### Immune Status Toward Pneumococcal Serotypes Before PCV13 Vaccination

**Figure 1:**
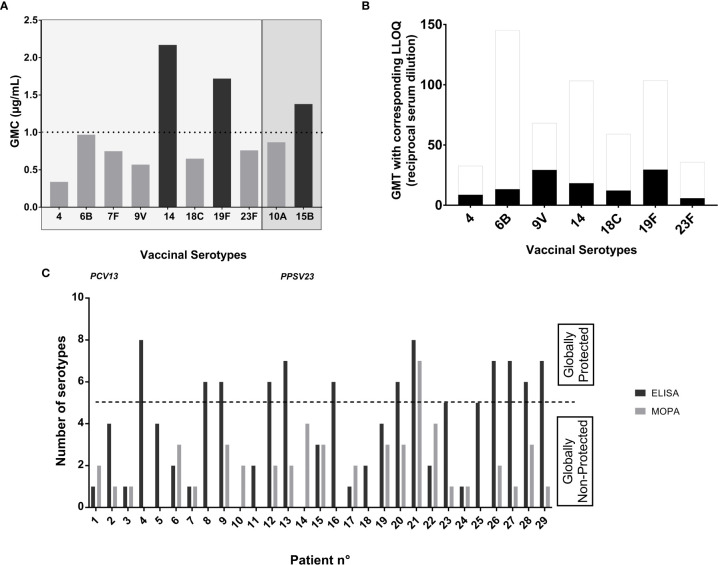
Antibodies concentrations and opsonization titers toward pneumococcal serotypes before PCV13 vaccination. **(A)** Seric IgG concentrations at baseline: Data are in geometric mean concentrations (GMC) (µg/mL) calculated from seric IgG concentrations in ELISA for each tested serotype. Protection was defined by an IgG concentration ≥1 μg/ml. The two serotypes 10A and 15B are not included in PCV13 vaccine and are represented here as controls. **(B)** Opsonization titers at baseline: Data are in geometric mean titers (GMT) (reciprocal serum dilution) calculated from opsonization titers (OT) in MOPA for each tested serotype. Protection was defined as an OT≥LLOQ. LLOQ are represented as white columns above the GMT in black columns for each serotype. **(C)** “Global protection” at baseline: Number of pneumococcal serotypes for which an IgG concentration ≥1 μg/ml tested by ELISA and an OT≥LLOQ by MOPA was achieved, for each patient. Patients developing a protection for at least five of the tested serotypes were considered as “globally protected” toward the serotypes included in the vaccine.

At baseline, only three serotypes (14, 19F and 15B) showed IgG GMC above the protective threshold by ELISA (**Figure 1A** **)** and none by analyses of GMT by MOPA (**Figure 1B**). Half of the patients (14/29) had specific IgG concentrations ≥1 µg/mL for at least five out of the eight tested serotypes, and were therefore considered as already “globally protected”. However, functional IgG activity analyzed by MOPA was low toward every serotype and only one patient (patient n°21 with IgG subclass deficiency, and previous PPSV23 vaccination) was considered as “globally protected” by MOPA before PCV13 vaccination (**Figure 1C**).

### Immune Response One-Month After PCV13 Vaccination

**Figure 2:**
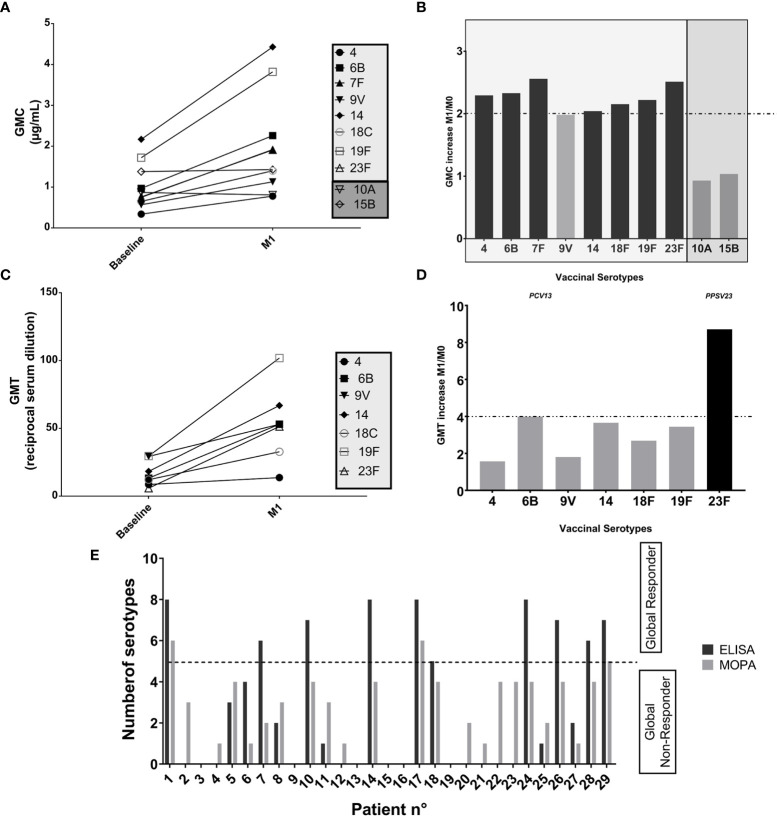
Impact of PCV13 vaccination: evaluation of the immune response based upon ELISA and MOPA analyses toward pneumococcal serotypes, one month after vaccination. **(A)** Evolution of seric IgG concentrations at M1: Data are in geometric mean concentrations (GMC, µg/mL) and its 95% confidence limits calculated from seric IgG concentrations (ELISA) for each tested serotype. The two serotypes 10A and 15B are not included in PCV13 vaccine and are represented here as controls. **(B)** Response evaluated by seric IgG concentration-increase, against each tested vaccine serotype: Response toward a serotype was defined as a two-fold increase from baseline in serotype specific IgG concentration at one month after vaccination based on ELISA analyses (ratio of GMC at M1 and GMC at M0 for each serotype). The two serotypes 10A and 15B are not included in PCV13 vaccine and are represented here as controls. **(C)** Evolution of opsonization titers at M1: Data are in geometric mean titers (GMT, reciprocal serum dilution) and its 95% confidence limits calculated from opsonization titers (MOPA), for each tested serotype. **(D)** Response evaluated by opsonization titers-increase, against each tested vaccine serotype: Response toward a serotype was defined as a four-fold increase in OT from baseline by MOPA at one month after vaccination. Response was defined as an at least four-fold increase from baseline in OT (ratio of GMT at M1 and GMT at M0 for each serotype) based on MOPA analyses. **(E)** “Global Response” at M1: Number of serotypes for which a two-fold increase from baseline in IgG concentration (ELISA) and an at least four-fold increase from baseline in OT (MOPA) was achieved, for each patient. Patients developing a response for at least five of the tested serotypes were considered as “global responders” to the vaccine.

One month after PCV13 vaccination, we observed an increase in GMC of antibodies directed against all serotypes (**Figure 2A**). A two-fold increase in IgG GMC was reached for every serotype except for 9V (**Figure 2B**). A significant increase in GMT was also observed for almost all serotypes, except the serotype 4 (**Figure 2C**). A four-fold increase in GMT was obtained for only one serotype (23F) (**Figure 2D**).

Overall, one month after PCV13, 10/28 (35.7%) patients with complete data were considered as “global responders” based on ELISA analysis and 3/28 (10.7%) based upon MOPA evaluation (**Figure 2E**). Interestingly, the three “global responders” patients by MOPA (n°1, 17, 29) were all “global responders” by ELISA and none of them had previously received PPSV23.

There was no difference between “global responders” and other patients assayed by ELISA depending on demographic characteristics, medical history, deficiency type, nor memory B cells concentrations at baseline. In contrast, patients were less likely to become “global responders” if they were undergoing immunoglobulin replacement therapy (6; 60% *vs* 17; 94.4%, p=0.04) or if they had previously received PPSV23 (0 *vs* 8; 44.4%, p=0.03) (**Supplemental Table 1**).

### Protection After PCV13 Vaccination Against the Pneumococcal Serotypes Tested at M1, M6 and M12

**Figure 3:**
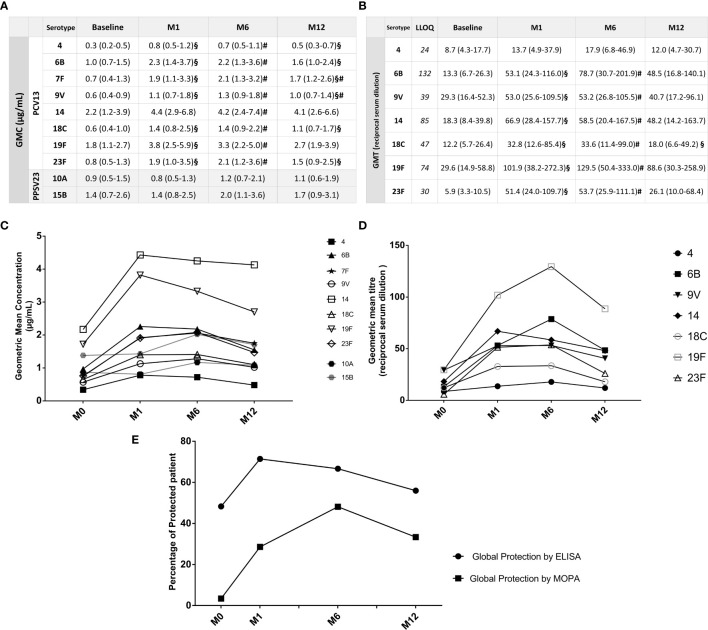
Durability of protection based upon ELISA and MOPA analyses. **(A)** IgG seric GMC against each tested vaccinal serotype, at baseline, M1, M6 and M12: Data are in geometric mean concentrations (µg/mL) and its 95% confidence limits calculated from seric IgG concentrations (ELISA), for each tested serotype. ^§^p < 0.05 when compared with previous concentration (Baseline/M1; M1/M6; M6/M12). ^#^p < 0.05 when compared with baseline. The two serotypes 10A and 15B are not included in PCV13 vaccine and are represented here as controls. **(B)** Opsonization titers GMT against each tested vaccinal serotype, at baseline, M1, M6 and M12: Data are in geometric mean titers (GMT) (reciprocal serum dilution) and its 95% confidence limits calculated from OT (MOPA), for each tested serotype. ^§^p < 0.05 when compared with previous concentration (Baseline/M1; M1/M6; M6/M12). ^#^p < 0.05 when compared with baseline. **(C)** Evolution of IgG seric GMC through time: Representation of the evolution in time of the Geometric Mean Concentrations (GMC) of IgG (ELISA) for each tested serotype. The two serotypes 10A and 15B are not included in PCV13 vaccine and are represented here as controls. **(D)** Evolution of OT GMT through time: Representation of the evolution in time of the Geometric Mean Titers (GMT) of OT (MOPA) for each tested serotype. **(E)** Evolution of “Global protection” according to ELISA or MOPA analyses, through time: Percentage of “globally protected” patients among patients with complete data, based upon ELISA and MOPA analyses, at baseline and at one, six and twelve months after vaccination. Patients were considered as “globally” protected if IgG concentration was ≥ 1µg/mL for ≥5 of the serotypes according to ELISA analysis and if OT ≥LLOQ for ≥5 of the serotypes based on MOPA evaluation.

One-month after PCV13 vaccination, GMC were above the protective threshold for every serotype except serotype 4. GMT were above their respective LLOQ for only three serotypes (9V, 19F, 23F) (**Figures 3A–D**). Overall, 71.4% of the patients (20/28) were considered as “globally protected” based on ELISA and 28.6% (8/28) based on MOPA (**Figure 3E**).

**Figure 4:**
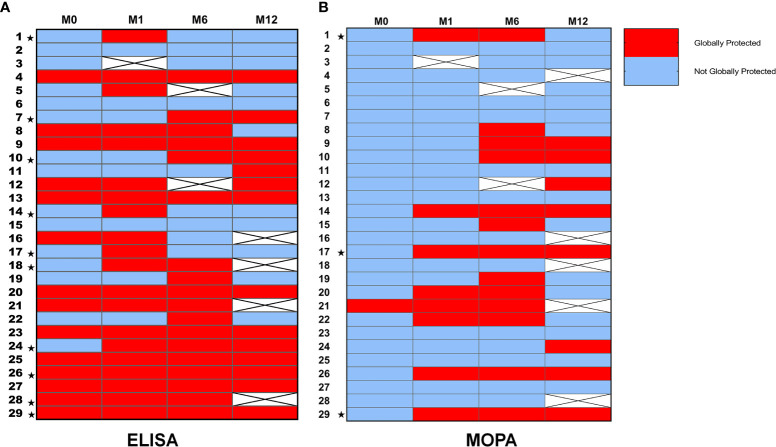
Global response and global protection for every patient. **(A)** Global protection and global response according to ELISA analyses: Individual data for “global protection” are represented in this heatmap. “Global protection” based upon ELISA analyses was defined if ≥5/8 of serotypes-specific IgG concentrations were ≥1µg/mL. Patients considered as “globally responders” at one month are signaled with the ★symbol. “Global Response” based upon ELISA analyses was defined if ≥5/8 of serotypes-specific IgG concentration had at least a two-fold increase one month after immunization. Missing data are represented as crossed white squares. **(B)** Global protection and global response according to MOPA analyses: Individual data for “global protection” are represented in this heatmap. “Global protection” based upon MOPA evaluation was defined if OT≥LLOQ was achieved for ≥5/7 of tested serotypes. Patients considered as “global responders” at one month are signaled with the ★symbol. “Global Response” based upon MOPA evaluation was defined if OT had an at least four-fold increase from baseline one month after vaccination for ≥5/7 of tested serotypes. Missing data are represented as crossed white squares.

Six months post-vaccination, GMC for most serotypes were stable when compared with M1 but significantly decreased (except for serotypes 7F and 9V) one year after PCV13 vaccination. Analyses by MOPA showed an increase in GMT compared to baseline for every serotype (except serotype 4) followed by a decrease to baseline at M12 for every serotype (**Figures 3A–D**). Overall, “global protection” evaluated by ELISA was stable at M6 (18/27, 66.7%) but decreased one-year post vaccination (14/25, 56.0%). Analyses by MOPA also showed a high rate of patients “globally protected” at M6 (13/27, 48.2%), and a fewer part at M12 (8/24, 33.3%) (**Figure 3E**). The **Figure 4** recapitulates all individual status regarding protection by ELISA and MOPA from baseline to one-year post vaccination.

Importantly, neither grade 2, 3 and 4 side effects nor documented pneumococcal infection were observed in the population during all the study.

### Factors Associated With Protection Against the Pneumococcal Serotypes Tested at Baseline and at M1, M6 and M12 After PCV13 Vaccination

At baseline, patients undergoing immunoglobulin replacement therapy were more likely to be already “globally protected” (p=0.02), as were those with higher serum IgG1 concentrations at diagnosis (3.54 ± 0.70 g/L *vs* 2.95 ± 0.99 g/L, p=0.049) and with older age (mean age in protected patients: 51.8 ± 14.6 *vs* 37.7 ± 14.5 in non-protected patients, p=0.02) according to ELISA analysis (**Supplemental Table 2**). There was no significant difference according to previous PPSV23 vaccination. The only “globally protected” patient according to MOPA analysis at baseline was a patient with IgG subtype deficiency, undergoing Ig replacement therapy and previously vaccinated with PPSV23.

At M1, all patients already “globally protected” at baseline were still “globally protected” according to ELISA (**Figure 4**). We found no significant difference in clinical characteristics between protected and non-protected patients at M1. The only immunological factor associated with “global protection” at M1 by ELISA was higher serum IgG1 concentrations at diagnosis (3.56 ± 0.70 g/L *vs* 2.58 ± 1.03 g/L, p=0.02) as observed at baseline (**Supplemental Table 3**). According to MOPA results, “global protection” at M1 was associated with “global response” to PCV13 (p=0.02). Moreover, higher serum total IgG concentrations at immunodeficiency diagnosis were associated with a better chance to reach “global protection” at M1 by MOPA (5.40 ± 0.94 *vs* 3.71 ± 1.86, p=0.02, data not shown).

At M6, patients with IgG subclass deficiency and those undergoing immunoglobulin replacement therapy were more likely to be protected as assayed by ELISA (**Supplemental Table 4**). There was no significant difference between protected and not protected patients at M6 as assayed by MOPA (data not shown).

The maintenance of “global protection” at M12 after vaccination, according to ELISA results, was associated with higher serum IgG2 concentrations at diagnosis (1.30 ± 0.71 g/L *vs* 0.63 ± 0.53 g/L, p=0.04; AUC = 0.78) (**Supplemental Table 5**). We found no other significant differences between protected and non-protected patients at M12 as tested by ELISA and by MOPA (data not shown).

## Discussion

In this study, we examined for the first time the immunogenicity of the PCV13 vaccine in patients with humoral PID. Humoral response one month after vaccination was reached for a third of the patients, allowing the protection of 71.4% of the patients. Moreover, improvement in functional activity of anti-pneumococcal antibodies was observed in a third of the patients. One-year post-vaccination, 56.0% and 33% of the patients were still considered as globally protected as assessed by ELISA and MOPA respectively.

We would like to highlight here that a large number of the patients already displayed high levels of anti-pneumococcal IgG before PCV13 vaccination and could be considered as already “globally protected”. By contrast, a study on PCV13 immunity in patients with systemic lupus performed with similar methods reported low anti-pneumococcal antibodies levels at baseline in this latter population (24). In our immunocompromised population, protection before PCV13 was observed especially in older patients (probably because of numerous past infectious events) and in patients receiving immunoglobulin replacement therapy, that contain blood donors derived-anti-pneumococcal antibodies (15, 25). Nevertheless, immunoglobulins of most patients displayed poor opsonophagocytic activities since only one patient was “globally protected” according to MOPA at baseline.

Regarding the immune response to PCV13 at one month, only a third of the patients (10/28) was able to produce significant amounts of specific anti-pneumococcal IgG. Even fewer (3/28) could be defined as “global responders” by MOPA, suggesting again their poor ability to produce IgG with high opsonophagocytic activities. This disparity between quantitative and functional results has not been reported in other populations after PCV13 vaccine, to our knowledge (20, 26). Indeed, patients with smoldering multiple myeloma developed a better global response one-month after PCV13 as compared to our population both for IgG concentrations and OT (global response achieved in respectively 60% and 40% of the patients with myeloma) (20). We can hazard a guess that antibodies produced by PID patients may not display sufficient avidity to elicit optimal complement activation (27). However, an important heterogeneity was noted among PID patients. Despite our first hypotheses, vaccine response was not influenced by immunodeficiency type, nor lymphocytes subpopulation repartition. By contrast, patients receiving immunoglobulin substitution or with prior PPSV23 vaccination were less likely to be responders. These latter patients could display a more severe immunodeficiency. Moreover, prior PPSV23 vaccination may favor hyporesponsiveness. Several studies in healthy adults and children reported reduced responses following repeated anti-pneumococcal vaccinations with polysaccharides consistent with hyporesponsiveness (28). The mechanisms are not fully understood but may involve immune tolerance and depletion of the B memory-cell pool (28, 29).

Disease prevention represents a better criterion than vaccine response for prophylactic vaccines like PCV13. In our approach, we used pre-existing immunological criteria to define vaccine-induced protection against pneumococcal infection. Although these criteria cannot substitute clinical criteria, they have been validated by other teams for PCV13 immunity analyses (20, 30, 31). In our cohort, protection one month after vaccination defined by ELISA’s results was obtained for 60% of the patients with IgG subclass deficiency and only for 40% of CVID patients and was associated with higher serum IgG1 concentrations at diagnosis. Importantly, PCV13 was able to improve antibodies opsonophagocytic activity since protection measured by MOPA was obtained for 28.6% of the patients. By using a similar immunological approach, a study on ten patients with subclass deficiency reported that protection was reached in 80% regarding both IgG concentrations and functional activities at one month after PCV13 (26). Taken together, these results advocate for vaccine’s benefits in PID population. Moreover, these results suggest that protection tends to be more easily reached in patients with subclass deficiency than in patients with a more global humoral deficiency.

The durability of vaccine induced-protection is another crucial point to further adapt vaccination strategy, especially in patients with lifelong PID. Here, we observed a progressive decrease in the percentage of “globally protected” patients (71.4% and 28.6% at one-month, 66.7% and 48.2% at six months and 56.0% and 33.3% at one-year, as assessed by ELISA and MOPA respectively). Decrease in both IgG concentrations and opsonophagocytic activities has been observed in healthy older adults with PCV13 (32). In the case of smoldering multiple myeloma, the decrease in IgG concentrations and OT was faster than observed here as only 25% and 10% of the patients with myeloma remained protected at one-year according to ELISA and MOPA assays, respectively (20). These data could suggest that booster doses are necessary in immunocompromised patients and that the prime-boost strategy should be individually adapted, regarding immunodeficiency type and IgG subclass concentrations in PID. Indeed, we observed that patients with higher serum IgG2 concentrations at diagnosis were more likely to stay protected at one-year. These data substantiate a recent study in patients with systemic lupus, for whom higher IgG2 serum concentrations were associated with long-term protection 36 months after a prime-boost PCV13/PPSV23 vaccination (33). IgG2 are known to play a key-role during pneumococcal infections (34, 35).

In conclusion, this study based on immunological criteria demonstrates for the first time the benefit of PCV13 vaccine in certain patients with PID. Some factors associated with better chances to develop a good immune response, and a sustained protection toward vaccine-included pneumococcal serotypes have been unraveled such as PID type, serum IgG1 and IgG2 levels at PID diagnosis. These results were obtained in a relatively small cohort of patients but if confirmed in larger studies, these factors could help physicians to better individually adapt the prime-boost anti-pneumococcal vaccine strategy in these patients with high risk of pneumococcal diseases.

Finally, this study opens new perspectives in the investigation of conjugate vaccine immunogenicity in patients with primary immunodeficiency. Indeed, the impact of past and booster polysaccharidic anti-pneumococcal vaccines needs to be further addressed. Moreover, new assays are being developed, allowing to explore specific antibody-antigen driven complement deposition, antibody dependent cellular cytotoxic and antibody dependent phagocytosis (36, 37). These assays could be of interest to explore non-neutralizing antibodies’ functions and therefore to better assess the correlates of protection in larger trials. Indeed, this new strategy allows to quantify antibody-dependent phagocytosis (by monocytes or neutrophiles) or antibody-dependent complement deposition for a specific antigen in a sample sparing-fashion. Likewise, Antibody-Dependent Natural Killer cells Activation (ADNKA), already studied in Influenza or Ebola vaccine-induced immunity (38), could bring out new information in the understanding of pneumococcal conjugate vaccine immunogenicity as we know that NK cells can be of importance in the control of bacterial burden in pneumococcal disease (39).

## Data Availability Statement

The raw data supporting the conclusions of this article will be made available by the authors, without undue reservation.

## Ethics Statement

The studies involving human participants were reviewed and approved by institutional review board, Reims University Hospital. The patients/participants provided their written informed consent to participate in this study.

## Author Contributions

AR made substantial contribution to acquisition of all clinical data, analyses and interpretation of data an in drafting the article. MB made substantial contribution to acquisition of all experimental data, analyses and interpretation of data and in drafting the article. MH made substantial contribution to statistical analyses and in revising the manuscript critically. QM made substantial contribution to acquisition of all clinical data and in revising the manuscript critically. CB made substantial contribution to conception of the study and to ethical issues and in revising the manuscript critically. DG, RN, FB, and AS made substantial contributions to conception and design, interpretation of data, and in revising the manuscript critically for important intellectual content. All authors contributed to the article and approved the submitted version.

## Funding

This work was supported by a grant from the “Société de Pathologie Infectieuse de Langue Française” (SPILF) in 2015.

## Conflict of Interest

The authors declare that the research was conducted in the absence of any commercial or financial relationships that could be construed as a potential conflict of interest.
