# Supplementary material for: The 13-Valent Pneumococcal Conjugate Vaccine Elicits Serological Response and Lasting Protection in Selected Patients With Primary Humoral Immunodeficiency
Source: Front Immunol. 2021 Jul 5;12:697128. doi: 10.3389/fimmu.2021.697128 (PMC8287634; doi:10.3389/fimmu.2021.697128)
Supplement: Supplementary file 1 [file Table_1.docx]

|  | **Responder M1**  **n=10** | **Non Responder M1**  **n=18** | **p** |
| --- | --- | --- | --- |
| **Age (mean±ST)** | 36.3±15.4 | 49.4±15.1 | 0.06 |
| **Age at diagnostic (mean±ST)** | 33.6±14.0 | 39.6±18.6 | 0.37 |
| **Male n(%)** | 1 (10.0) | 7 (38.9) | 0.19 |
| **Subclass n(%)** | 6 (60.0) | 8 (44.4) | 0.69 |
| **CVID n(%)** | 4 (40.0) | 10 (55.6) |  |
| **Ig replacement therapy n(%)** | 6 (60.0) | 17 (94.4) | **0.04** |
| **Prior anti-pneumococcal vaccination n(%)** | 0 | 8 (44.4) | **0.03** |
| **Prior invasive pneumococcal infection n(%)** | 0 | 3 (16.7) | 0.53 |
| **IgG (mean±ST)** | 4.56±1.49 | 3.99±1.98 | 0.52 |
| **IgG1 (mean±ST)** | 3.24±0.92 | 3.28±0.94 | 0.64 |
| **IgG2 (mean±ST)** | 1.11±0.76 | 1.17±0.72 | 1.00 |
| **IgG3 (mean±ST)** | 0.22±0.10 | 0.26±0.16 | 0.74 |
| **IgG4 (mean±ST)** | 0.14±0.13 | 0.12±0.09 | 0.84 |
| **IgA (mean±ST)** | 0.89±0.64 | 0.53±0.56 | 0.20 |
| **IgM (mean±ST)** | 0.78±0.53 | 0.52±0.45 | 0.15 |
| **Lymphocyte count (mean±ST)** | 1.65±0.36 | 1.58±0.71 | 0.47 |
| **CD19 lymphocyte (mean±ST)** | 257.60±129.39 | 183.09±140.40 | 0.11 |
| **Naive B cell (mean±ST)** | 201.60±137.33 | 135.17±116.41 | 0.21 |
| **Non-switched memory B cell (mean±ST)** | 28.31±18.76 | 30.65±30.82 | 0.63 |
| **Switched memory B-cell (mean±ST)** | 19.91±20.01 | 12.71±13.94 | 0.63 |
| **CD4 lymphocyte (mean±ST)** | 772.90±204.69 | 743.35±349.35 | 0.86 |
| **Naive T cell (mean±ST)** | 262.38±123.91 | 233.96±189.66 | 0.52 |

**Supplemental Table 1: Factors associated with “global response” at M1**

ST: Standard deviation.

CVID: Common Variable ImmunoDeficiency

Ig ponderal dosage in g/L: immunoglobulin ponderal dosage in serum at diagnosis when available or before immunoglobulin substitution initiation

Lymphocytes subpopulation (10^6^/L): immunophenotyping of the main B and T cell subpopulation in serum when available
