# Supplementary material for: The 13-Valent Pneumococcal Conjugate Vaccine Elicits Serological Response and Lasting Protection in Selected Patients With Primary Humoral Immunodeficiency
Source: Front Immunol. 2021 Jul 5;12:697128. doi: 10.3389/fimmu.2021.697128 (PMC8287634; doi:10.3389/fimmu.2021.697128)
Supplement: Supplementary file 2 [file Table_2.docx]

|  | **Protected M0**  **n=14** | **Non Protected M0**  **n=15** | **p** |
| --- | --- | --- | --- |
| **Age (mean±ST)** | 51.8±14.6 | 37.7±14.5 | **0.02** |
| **Age at diagnostic (mean±ST)** | 34.5±28.2 | 25.8±34.8 | 0.38 |
| **Male n(%)** | 4 (28.6) | 4 (26.7) | 1.00 |
| **Subclass n(%)** | 9 (64.3) | 5 (33.3) | 0.14 |
| **CVID n(%)** | 5 (35.7) | 10 (66.7) |  |
| **Ig replacement therapy n(%)** | 14 (100.0) | 9 (60.0) | **0.02** |
| **Prior anti-pneumococcal vaccination n(%)** | 4 (28.6) | 5 (33.3) | 1.00 |
| **Prior invasive pneumococcal infection n(%)** | 0 | 3 (20.0) | 0.22 |
| **IgG (mean±ST)** | 4.51±2.08 | 3.86±1.46 | 0.07 |
| **IgG1 (mean±ST)** | 3.54±0.70 | 2.99±0.99 | **0.049** |
| **IgG2 (mean±ST)** | 1.42±0.72 | 0.84±0.61 | 0.06 |
| **IgG3 (mean±ST)** | 0.23±0.16 | 0.25±0.10 | 0.33 |
| **IgG4 (mean±ST)** | 0.16±0.12 | 0.08±0.08 | 0.10 |
| **IgA (mean±ST)** | 0.71±0.61 | 0.58±0.61 | 0.63 |
| **IgM (mean±ST)** | 0.70±0.58 | 0.71±0.79 | 0.78 |
| **Lymphocyte count (mean±ST)** | 1.56±0.61 | 1.69±0.60 | 0.64 |
| **CD19 lymphocyte (mean±ST)** | 178.49±108.63 | 229.38±162.63 | 0.57 |
| **Naive B cell (mean±ST)** | 116.43±80.71 | 187.51±155.13 | 0.33 |
| **Non-switched memory B cell (mean±ST)** | 38.12±30.23 | 22.40±19.89 | 0.34 |
| **Switched memory B-cell (mean±ST)** | 19.95±18.96 | 10.22±10.80 | 0.31 |
| **CD4 lymphocyte (mean±ST)** | 779.20±309.97 | 754.15±298.70 | 0.72 |
| **Naive T cell (mean±ST)** | 272.75±189.21 | 241.76±162.97 | 0.78 |

**Supplemental Table 2: Factors associated with “global protection” at baseline**

ST: Standard deviation.

CVID: Common Variable ImmunoDeficiency

Ig replacement therapy: Intravenous or subcutaneous immunoglobulin

Ig ponderal dosage in g/L: immunoglobulin ponderal dosage in serum at diagnosis when available or before immunoglobulin substitution initiation

Lymphocytes subpopulation (10^6^/L): immunophenotyping of the main B and T cell subpopulation in serum, when available
