# Supplementary material for: The 13-Valent Pneumococcal Conjugate Vaccine Elicits Serological Response and Lasting Protection in Selected Patients With Primary Humoral Immunodeficiency
Source: Front Immunol. 2021 Jul 5;12:697128. doi: 10.3389/fimmu.2021.697128 (PMC8287634; doi:10.3389/fimmu.2021.697128)
Supplement: Supplementary file 3 [file Table_3.docx]

|  | **Protected M1**  **n=20** | **Non Protected M1**  **n=8** | **p** |
| --- | --- | --- | --- |
| **Age (mean±ST)** | 46.2±16.9 | 41.1 ±14.8 | 0.40 |
| **Age at diagnostic (mean±ST)** | 27.7±37.0 | 35.6±14.3 | 0.98 |
| **Male n(%)** | 5 (25.0) | 3 (37.5) | 0.65 |
| **Subclass n(%)** | 12 (60.0) | 2 (25.0) | 0.21 |
| **CVID n(%)** | 8 (40.0) | 6 (75.0) |  |
| **Ig replacement therapy n(%)** | 16 (80.0) | 7 (87.5) | 1.00 |
| **Prior anti-pneumococcal vaccination n(%)** | 5 (25.0) | 3 (37.5) | 0.65 |
| **Prior invasive pneumococcal infection n(%)** | 1 (5.0) | 2 (25.0) | 0.19 |
| **IgG (mean±ST)** | 4.42±1.87 | 3.63±1.63 | 0.14 |
| **IgG1 (mean±ST)** | 3.56±0.70 | 2.58±1.03 | **0.02** |
| **IgG2 (mean±ST)** | 1.28±0.70 | 0.77±0.70 | 0.20 |
| **IgG3 (mean±ST)** | 0.25±0.14 | 0.23±0.13 | 0.97 |
| **IgG4 (mean±ST)** | 0.14±0.11 | 0.07±0.08 | 0.12 |
| **IgA (mean±ST)** | 0.79±0.63 | 0.32±0.37 | 0.08 |
| **IgM (mean±ST)** | 0.67±0.53 | 0.46±0.35 | 0.42 |
| **Lymphocyte count (mean±ST)** | 1.55±0.55 | 1.73±0.73 | 0.75 |
| **CD19 lymphocyte (mean±ST)** | 202.11±122.59 | 232.70±180.76 | 1.00 |
| **Naive B cell (mean±ST)** | 141.47±111.41 | 196.69±154.78 | 0.46 |
| **Non-switched memory B cell (mean±ST)** | 33.37±28.00 | 21.76±23.71 | 0.46 |
| **Switched memory B-cell (mean±ST)** | 19.46±17.65 | 5.51±5.40 | 0.07 |
| **CD4 lymphocyte (mean±ST)** | 759.54±288.63 | 742.04±345.02 | 0.80 |
| **Naive T cell (mean±ST)** | 279.55±173.38 | 168.74±128.97 | 0.29 |

**Supplemental Table 3: Factors associated with “global protection” at M1**

ST: Standard deviation.

CVID: Common Variable ImmunoDeficiency

Ig ponderal dosage in g/L: immunoglobulin ponderal dosage in serum at diagnosis when available or before immunoglobulin substitution initiation

Lymphocytes subpopulation (10^6^/L): immunophenotyping of the main B and T cell subpopulations in serum
