# Supplementary material for: The 13-Valent Pneumococcal Conjugate Vaccine Elicits Serological Response and Lasting Protection in Selected Patients With Primary Humoral Immunodeficiency
Source: Front Immunol. 2021 Jul 5;12:697128. doi: 10.3389/fimmu.2021.697128 (PMC8287634; doi:10.3389/fimmu.2021.697128)
Supplement: Supplementary file 4 [file Table_4.docx]

|  | **Protected M6**  **n=18** | **Non Protected M6**  **n=9** | **p** |
| --- | --- | --- | --- |
| **Age (mean±ST)** | 45.0±16.3 | 44.9±14.8 | 1.0 |
| **Age at diagnostic (mean±ST)** | 36.8±17.2 | 17.8±49.7 | 0.7 |
| **Male n(%)** | 4 (22.2) | 4 (44.4) | 0.37 |
| **Subclass n(%)** | 12 (66.7) | 2 (22.2) | **0.046** |
| **CVID n(%)** | 6 (33.3) | 7 (77.8) |  |
| **Ig replacement therapy n(%)** | 17 (94.4) | 4 (44.4) | **0.008** |
| **Prior anti-pneumococcal vaccination n(%)** | 5 (27.8) | 3 (33.3) | 1.0 |
| **Prior invasive pneumococcal infection n(%)** | 1 (5.6) | 1 (11.1) | 1.0 |
| **IgG (mean±ST)** | 4.53±1.78 | 4.14±1.32 | 0.17 |
| **IgG1 (mean±ST)** | 3.23±0.81 | 3.26±1.11 | 1.0 |
| **IgG2 (mean±ST)** | 1.30±0.74 | 0.78±0.57 | 0.09 |
| **IgG3 (mean±ST)** | 0.24±0.16 | 0.23±0.07 | 0.8 |
| **IgG4 (mean±ST)** | 0.15±0.11 | 0.07±0.07 | 0.13 |
| **IgA (mean±ST)** | 0.77±0.66 | 0.52±0.45 | 0.4 |
| **IgM (mean±ST)** | 0.68±0.48 | 0.84±1.03 | 0.8 |
| **Lymphocyte count (mean±ST)** | 1.63±0.57 | 1.59±0.71 | 0.7 |
| **CD19 lymphocyte (mean±ST)** | 184.59±108.52 | 228.43±191.45 | 0.9 |
| **Naive B cell (mean±ST)** | 121.52±88.61 | 194.56±175.21 | 0.4 |
| **Non-switched memory B cell (mean±ST)** | 33.80±28.22 | 19.28±18.44 | 0.3 |
| **Switched memory B-cell (mean±ST)** | 19.32±18.65 | 8.65±5.18 | 0.5 |
| **CD4 lymphocyte (mean±ST)** | 807.83±272.66 | 674.76±362.03 | 0.2 |
| **Naive T cell (mean±ST)** | 284.20±175.57 | 218.70±179.60 | 0.4 |

**Supplemental Table 4: Factors associated with “global protection” at M6**

ST: Standard deviation.

CVID: Common Variable ImmunoDeficiency

Ig ponderal dosage in g/L: immunoglobulin ponderal dosage in serum at diagnosis when available or before immunoglobulin substitution initiation

Lymphocytes subpopulation (10^6^/L): immunophenotyping of the main B and T cell subpopulations in serum
