# Supplementary material for: The 13-Valent Pneumococcal Conjugate Vaccine Elicits Serological Response and Lasting Protection in Selected Patients With Primary Humoral Immunodeficiency
Source: Front Immunol. 2021 Jul 5;12:697128. doi: 10.3389/fimmu.2021.697128 (PMC8287634; doi:10.3389/fimmu.2021.697128)
Supplement: Supplementary file 5 [file Table_5.docx]

|  | **Protected M12**  **n=14** | **Non Protected M12**  **n=11** | **p** |
| --- | --- | --- | --- |
| **Age (mean±ST)** | 48.0±16.1 | 40.8±15.3 | 0.18 |
| **Age at diagnostic (mean±ST)** | 41.1±17.4 | 33.3±15.5 | 0.26 |
| **Male n(%)** | 2 (14.3) | 5 (45.5) | 0.18 |
| **Subclass n(%)** | 7 (50.0) | 3 (27.3) | 0.41 |
| **CVID n(%)** | 7 (50.0) | 8 (72.7) |  |
| **Ig replacement therapy n(%)** | 12 (85.7) | 7 (63.6) | 0.35 |
| **Prior anti-pneumococcal vaccination n(%)** | 2 (14.3) | 6 (54.6) | 0.08 |
| **Prior invasive pneumococcal infection n(%)** | 0 | 3 (27.3) | 0.07 |
| **IgG (mean±ST)** | 4.20±2.10 | 3.70±1.52 | 0.27 |
| **IgG1 (mean±ST)** | 3.40±0.87 | 3.06±1.10 | 0.25 |
| **IgG2 (mean±ST)** | 1.30±0.71 | 0.63±0.53 | **0.04** |
| **IgG3 (mean±ST)** | 0.24±0.17 | 0.23±0.09 | 0.65 |
| **IgG4 (mean±ST)** | 0.15±0.13 | 0.08±0.07 | 0.28 |
| **IgA (mean±ST)** | 0.64±0.66 | 0.59±0.60 | 0.85 |
| **IgM (mean±ST)** | 0.65±0.52 | 0.64±0.91 | 0.41 |
| **Lymphocyte count (mean±ST)** | 1.54±0.52 | 1.62±0.64 | 0.89 |
| **CD19 lymphocyte (mean±ST)** | 190.52±113.59 | 214.89±184.18 | 0.97 |
| **Naive B cell (mean±ST)** | 137.35±98.39 | 174.43±172.72 | 0.95 |
| **Non-switched memory B cell (mean±ST)** | 28.82±21.49 | 22.02±18.12 | 0.76 |
| **Switched memory B-cell (mean±ST)** | 13.91±18.51 | 11.92±8.78 | 0.50 |
| **CD4 lymphocyte (mean±ST)** | 770.66±275.73 | 696.33±341.87 | 0.47 |
| **Naive T cell (mean±ST)** | 230.11±172.02 | 249.00±171.58 | 0.92 |

**Supplemental Table 5: Factors associated with “global protection” at M12**

ST: Standard deviation. CVID: Common Variable ImmunoDeficiency

Ig replacement therapy: Intravenous or subcutaneous immunoglobulin

Ig ponderal dosage in g/L: immunoglobulin ponderal dosage in serum at diagnosis when available or before immunoglobulin substitution initiation

Lymphocytes subpopulation (10^6^/L): immunophenotyping of the main B and T cell subpopulations in serum
